# Ethical analysis in HTA of complex health interventions

**DOI:** 10.1186/s12910-016-0099-z

**Published:** 2016-03-22

**Authors:** Kristin Bakke Lysdahl, Wija Oortwijn, Gert Jan van der Wilt, Pietro Refolo, Dario Sacchini, Kati Mozygemba, Ansgar Gerhardus, Louise Brereton, Bjørn Hofmann

**Affiliations:** Centre for Medical Ethics, University of Oslo, Oslo, Norway; ECORYS Netherlands BV, Rotterdam, The Netherlands; Donders Institute for Brain, Cognition and Behavior, Radboud University Medical Centre, Nijmegen, The Netherlands; Athena Institute for Innovation in the Health and Life Sciences, VU University Amsterdam, Amsterdam, The Netherlands; Institute of Bioethics, “A. Gemelli” School of Medicine, Università Cattolica del Sacro Cuore, Rome, Italy; Department of Health Services Research, Institute of Public Health and Nursing Research, and Health Sciences, University of Bremen, Bremen, Germany; Health Sciences Bremen, Bremen, Germany; ScHARR, University of Sheffield, Sheffield, UK; The Norwegian University for Science and Technology, Gjøvik, Norway

**Keywords:** Health technology assessment, bioethics, complex health intervention

## Abstract

**Background:**

In the field of health technology assessment (HTA), there are several approaches that can be used for ethical analysis. However, there is a scarcity of literature that critically evaluates and compares the strength and weaknesses of these approaches when they are applied in practice. In this paper, we analyse the applicability of some selected approaches for addressing ethical issues in HTA in the field of complex health interventions. Complex health interventions have been the focus of methodological attention in HTA. However, the potential methodological challenges for ethical analysis are as yet unknown.

**Methods:**

Six of the most frequently described and applied ethical approaches in HTA were critically assessed against a set of five characteristics of complex health interventions: multiple and changing perspectives, indeterminate phenomena, uncertain causality, unpredictable outcomes, and ethical complexity. The assessments are based on literature and the authors’ experiences of developing, applying and assessing the approaches.

**Results:**

The Interactive, participatory HTA approach is by its nature and flexibility, applicable across most complexity characteristics. Wide Reflective Equilibrium is also flexible and its openness to different perspectives makes it better suited for complex health interventions than more rigid conventional approaches, such as Principlism and Casuistry. Approaches developed for HTA purposes are fairly applicable for complex health interventions, which one could expect because they include various ethical perspectives, such as the HTA Core Model® and the Socratic approach.

**Conclusion:**

This study shows how the applicability for addressing ethical issues in HTA of complex health interventions differs between the selected ethical approaches. Knowledge about these differences may be helpful when choosing and applying an approach for ethical analyses in HTA. We believe that the study contributes to increasing awareness and interest of the ethical aspects of complex health interventions in general.

## Background

Ethics has been on the health technology assessment (HTA) agenda since the inception of HTA. This is reflected in the definition of HTA: “a multidisciplinary process that summarises information about the medical, social, economic and ethical issues related to the use of a health technology in a systematic, transparent, unbiased, robust manner. Its aim is to inform the formulation of safe, effective, health policies that are patient focused and seek to achieve best value” [1]. Ethics should be integrated in HTA for a number of reasons. In HTA, the merits of health technologies are evaluated for the purpose of informing decision making in health care. This is a normative issue because health technologies and health care have a moral intention to help people, by improving health status, relieving pain etc., i.e. promoting a good life [2]. The core issues in HTA, such as safety and effectiveness, also raises moral questions about how these issues should be defined and measured, e.g. the limits for safety [2]. Moreover, including ethics can make HTA more efficient and the subsequent decisions more informed and accountable, thereby avoiding the HTA results being in conflict with social and moral values [2]. Cochlear Implants (CI) for children is a well know example where there are conflicting values [3], which demonstrate the importance of including ethics in HTA. A reason for the controversy was that CI can be seen as a remedy of a medical condition (deafness) or a threat against the Sign Language as a natural language in the Deaf community.

Over the years, a series of methods for ethical analysis in HTA have been developed and suggested [4]. However, there has not been a general acceptance for incorporating ethics in the HTA process until recently [5, 6], and ethics is still “rarely addressed explicitly in HTA reports” [7]. A survey of HTA reports published in the period 2003 to 2006 by HTA agencies in Canada, UK, Denmark and USA, showed that ethical, social and organizational issues, in addition to clinical and economic evaluations, were only considered in 5 % of the (223) reports found [4]. One of the many possible reasons for this [8] may be that the methods generally need further development to be feasible to the users. According to Assasi et al. [4], we lack studies that critically evaluate the characteristics of the available ethical approaches. Another reason may be that, as health interventions become more complex, existing methods for addressing ethical issues in HTA are not suitable for such interventions.

Recently the methodological challenges associated with complex health interventions^1^ for systematic reviews and evidence synthesis in HTA have gained much interest [9]. Complexity is associated with uncertainty and unpredictability, and most health interventions are complex to some degree. Highly complex health interventions, such as lifestyle interventions and treatment for chronic diseases, pose particular challenges for HTA, e.g. in terms of heterogeneous study designs. However, little interest has been shown about how complex health interventions may cause possible methodological challenges for ethical analysis in HTA. The aim of this paper is to investigate how applicable existing ethical approaches are for addressing ethical issues in HTA of complex health interventions. The applicability assessment will indicate how the approaches can be amended or improved to better address ethical aspects of complex^2^ health interventions.

## Methods

(Bio)medical ethics is a multidisciplinary field, i.e. a field that involves a range of disciplines^3^ that investigate the subject matter of medicine, health care and the life sciences using a wide variety of methods [10]. Accordingly, a series of methods or approaches are relevant for ethical assessment in HTA. These are fairly well mapped out, e.g. identified in the HTA Core Model®,^4^ in surveys [6, 11] and a recent systematic review [4]. Additionally, the authors of this study have made several contributions to the field, are active participants in e.g. EUnetHTA, HTAi-INAHTA Special Interest Group on Ethical issues in HTA and have followed the field for several years. Not all approaches used to address ethical issues in HTA can be assessed and presented in this study. Therefore, only approaches that are frequently described and used in HTA reports and peer reviewed articles and approaches initially considered to have the potential for addressing ethical issues in complex interventions are included.^5^ Three general approaches in ethics used in HTA are considered: The four principles approach, Casuistry, and Wide Reflective Equilibrium (coherence analysis). Additionally, three HTA specific approaches are selected: Interactive, participatory HTA approaches (iHTA), the HTA Core Model®, and the Socratic approach.

To assess the included approaches, we used an established set of characteristics of complexity that have ethical relevance: 1) multiple and changing perspectives, 2) indeterminate phenomena, 3) uncertain causality, 4) unpredictable outcomes, and 5) ethical complexity.^6^ These five characteristics are explained in Table 1, where some implications for ethical analyses in HTA are also given. The development of the set of ethically relevant complexity characteristics is mainly based on a) publications by the UK Medical Research Council [12], b) a recent paper by Petticrew and colleagues [9], and c) our deliberations about additional ethics specific characteristics, which is described elsewhere.^7^

**Table 1:** Characteristics of complexity, short explanations and implications for ethics analysis

Characteristic	Short explanation	Implications for ethical analysis in HTA^a^
Multiple and changing perspectives	The variety of perspectives is caused by the many components (social, material, theoretical, and procedural [51]), actors, stakeholders, organisational levels involved. Additionally, these are interconnected and interacting.	Address the variety of perspectives (typically the stakeholders’ interests and intentions), questions about normative implications of interconnectedness and interactions between actors/components, and moral questions related to control and decision making.
Indeterminate phenomena	The interventions or health condition cannot be strictly defined or delimited due to characteristics like flexibility, tailoring, self-organization, adaptivity, and evolution over time.	Identify moral challenges related to indeterminacy of the intervention and/or the target medical condition(s). E.g. identify possible contradictory interpretations^b^ and alternative use of the intervention, and the justifications of these.
Uncertain causality	Factors like synergy between components, feedback loops, moderators and mediators of effect, context and the symbolic value of the intervention lead to uncertain causal pathways between intervention and outcome.	Address morally relevant issues related to methodological choices in the HTA itself. The uncertainties call for transparency and openness about the grounds for the choices and an integrative approach.
Unpredictable outcomes	The outcomes of the intervention may be many, variable, new, emerging and unexpected.	Address ethical challenges of handling outcome uncertainties, regarding outcome type, size, for whom/at what level, and at what time.
Ethical complexity	Interventions are especially ethically complex because of contradictions between basic ethical principles, or because fundamental moral or sociocultural values are at stake.	Reveal underlying norms and values, and elucidate possible contradicting principles or values (resolvability). Reveal potential fundamental ethical, social, cultural values at stake, and contribute to handling of conflicting concerns. Clarity of aim and scope of ethical analyses (conclusiveness and integration in HTA), and comprehensiveness and transparency of reporting are essential.

^a^Describing some obvious implications certainly does not exclude the possibility of other relevant implications

^b^The indeterminacy of complex interventions allows for interpretations in different, also contradictory, ways, (i.e. paradoxes need careful scrutiny and conciliation of interpretations to be resolved) [52]

Unfortunately, the literature reporting on ethics analysis of complex interventions is too small for a systematic review and an analysis of how suitable the various ethics approaches are for such interventions. Therefore, we were forced to analyse and discuss the various ethical approaches against a set of complexity characteristics.^8^

## Results and discussion

In the following text, we briefly present the selected approaches for addressing ethical issues in HTA before analysing and discussing their appropriateness for complex health interventions, according to the characteristics presented in Table 1. Table 2 gives an overview of the results of these analyses.

**Table 2:** Summarized assessment of the ethical approaches according to characteristics of complexity

	Aspects of complexity				
Ethical approach	Plurality of perspectives	Indeterminate phenomena	Uncertain causality	Unpredictable outcomes	Ethical complexity
Principlism	*Hardly applicable*: A limited number of perspectives are included, the implications of interactions between agents are partially included.	*Hardly applicable*: Questions related to indeterminacy are not addressed.	*Hardly applicable*: Data required by the approach indicates that methodological choices in the HTA process may be partially addressed.	*Hardly applicable:* Ethical issues of outcomes are addressed, but not the uncertainties in outcomes as such.	*Fairly applicable*: Conflicting of principles can be illuminated, but not always overcome (resolvability).
Casuistry	*Hardly applicable*: Analogues can provide solutions taking different perspectives into account, but may not be suitable for joining/synthesizing/com-promising perspectives, or to address interconnectedness/interactions.	*Hardly applicable:* Analogues can provide potential conceptions of indefinite phenomena, but there is a threat of over-simplification.	*Hardly applicable:* Analogues may address uncertainties. However, whether the analogues will handle relevant potential uncertainties cannot be predicted.	*Hardly applicable:* Analogues may address un-predictability, but it may also mask basic or dynamic challenges, such as unpredictable outcomes.	*Fairly applicable*: Casuistry is excellent for finding solutions to morally challenging problems. However, Casuistry does not provide solutions to genuine paradoxes and aporias. It may be useful to highlight them, though.
Wide Reflective Equilibrium, (coherence analysis)	*Fairly applicable*: WRE can take into account of multiple perspectives and differences in judgement of moral properties. Interaction between components may be addressed in the WRE process. Control and decision-making is issued by the aim of providing a coherent base for this.	*Applicable*: The moral implications of the indeterminacy of the intervention or condition can be revealed and explored in discussions towards equilibrium.	*Fairly applicable*: Do not address moral issues related to methodological choices in HTA in general, but recognises the uncertainties from context dependency and the importance of taking this in to account.	*Fairly applicable*: Can accommodate different views of what constitute relevant end points. Unexpected outcomes may be interpreted as disruption of the equilibrium, calling for a renewed debate.	*Fairly applicable*: WRE can reveal fundamental values at stake, take value conflicts into account, elucidate contractions and inform about their resolvability. The aim of WRE is clear, but quality of reporting is not explicitly addressed.
Interactive, participatory HTA approaches (iHTA)	*Applicable*: iHTA is pre-eminently suited to take into account a variety of perspectives, and interaction between actors.	*Applicable*: Indeterminacy of a technology and its use is acknowledged.	*Applicable*: Stakeholder involvement in the assessment process facilitates addressing ethical challenges in methodological choices.	*Fairly applicable*: The approach is likely to increase the range of outcomes taken into account, which indicates that the ethical challenges of this unpredictability are also addressed.	*Fairly applicable*: Stakeholders may reveal fundamental moral or socio-cultural values involved, and may elucidate the resolvability of possible contradicting principles/values.
The HTA Core Model®	*Fairly applicable*: Different perspectives are included through stakeholder involvement and cooperation with experts in other HTA-areas. Interactions/interrelations are not specified or related to ethical implications.	*Hardly applicable:* Defining the technology and target group is addressed in another domain of the model. Ethical implications of indeterminacy of technology/condition, are not addressed, but an illustration of ethical relevance of defining the target group is given.	*Fairly applicable*: Morally relevant issues related to methodological choices are addressed in the introduction to the core model, and to some extent in the ethics domain. Factors contributing to uncertain causality are not specifically included, but context is indirectly considered though context dependent values.	*Applicable:* Outcome uncertainties are addressed in the “beneficence/non-maleficence” issue, and in some other parts of the model.	*Fairly applicable:* Some fundamental values are directly addressed, others may be revealed by stakeholder involvement, which also may reveal contradicting principles/values. The contribution to handling conflicting concerns is limited. The (common) reporting structure contributes to transparency.
The Socratic approach	*Fairly applicable*: Identifies actors and stakeholders, and their perspectives, interest etc. Normative implications of interactions between agents (and components in general) are partly covered. Decision-making and responsibilities are also touched upon.	*Fairly applicable*: Provides a means for exploring various definitions/under-standing of the interventions. The moral impact of indeterminacy is not directly addressed, but may be illuminated through related questions.	*Applicable*: Morally relevant methodological choices in HTA are well addressed, which can contribute to an improvement in taking causal pathway uncertainties into account.	*Fairly applicable*: Variety in outcomes is not specifically addressed, but rather a series of moral question about different potential outcomes.	*Fairly applicable:* Reveals fundamental values and contribute to elucidate contradictions. The clear descriptive aim limits the contribution to handling conflicting concerns and contradictions. Comprehensiveness and transparency in reporting is emphasised.

### The four principle approach

The most frequently used form of Principlism (i.e. to apply principles to solve moral problems) addresses the four basic ethical principles: respect for autonomy, non-maleficence, beneficence and justice [13]. These principles have a prima facie nature, which means that the principle must be fulfilled unless it conflicts with an equal or stronger obligation. The principles constitute a basic framework and they need to be *specified* and *balanced* (i.e. the practical activity becomes that of specifying how the principles are to be used in specific situations and balancing the principles with the other competing moral principles). Principlism is a popular approach because it is simple and feasible. Its simplicity lies in the application of a stable set of ethical themes and concepts [14]. However, this simplicity also constitutes the major limitation of the approach: the risk of leaving out a series of values and perspectives. Furthermore, it has been questioned whether the (in this case four) principles (and only these) are universal [15].

#### Principlism and the characteristics of complexity

##### Variety of perspectives

Principlism is not designed as a participatory approach. It primarily takes into account physicians’ and patients’ perspectives, but tends to leave other stakeholder perspectives out. Society's perspective is taken into account (principle of justice), although in a top-down manner, i.e. according to the perspective of the user of the approach. Therefore, Principlism is only partially suited to address multiple perspectives and goals.

The four principles entail taking account of the view of physicians, patients and society. Therefore, the approach may encourage the user to think about normative issues and the implications of interactions/relationship between agents (particularly physician-patient relationships). Issues of control, decision-making and related responsibilities are not addressed in this approach.

##### Indeterminate phenomena

Respecting the principles of beneficence/non-maleficence requires a strict evaluation of the benefits and risks related to the use of technologies. However, the indeterminacy of the intervention and/or the target condition would make this type of evaluation difficult or even impossible. This means that Principlism is not very useful for identifying moral challenges related to indeterminate phenomena. This has been acknowledged e.g. in the case of nanotechnologies where the toxicological risk assessment for use of nanomaterials is difficult. Therefore, establishing the right balance between benefits for patients and society and risks of harms may be unclear [16].

##### Uncertain causality

As mentioned above, Principlism is not suitable for handling uncertainty. The symbolic value of an intervention is not addressed and the impact of context on outcome may be only partially recognized (subject to pure speculation).

Principlism does not directly include questions about morally relevant methodological choices in the HTA. Nevertheless, this approach requires data to be available on safety, efficacy/effectiveness (principles of non-maleficence/beneficence) and economic impact (principle of justice) related to the interventions. In this sense, Principlism may influence some choices in the HTA process.

##### Unpredictable outcomes

Principlism may encourage the user to think about some possible outcomes or consequences of an intervention, particularly benefits and harms (beneficence/non-maleficence), change of patient role (respect for autonomy) and effects on the distribution of health care resources (principle of justice). These issues are always addressed in a top-down manner, therefore they may be subject to pure speculation. Principlism is not suitable for handling uncertainties in outcomes (whatever the cause, type and size), e.g. if some kind of uncertainty itself may infer harm or challenge autonomy.

##### Ethical complexity

Principlism may be excellent for identifying possible contradictions (e.g. the conflict between autonomy and beneficence for single persons on the one hand, and the just distribution of resources and beneficence for society on the other). In addition, the “balancing activity” of Principlism may be a useful “tool” for solving possible conflicts and elucidating which moral norms should prevail. Even if identifying and balancing contradictions is apparently simple and feasible, it may not provide incontrovertible solutions to possible conflicts in practice, since there is no unified moral theory from which the four principles are derived. Besides, some contradictions may be overlooked due to the limited scope of the four principles approach.

In summary, Principlism does not seem well suited for addressing several aspects of complexity (see Table 2). Hence, where this is applied to complex interventions the approach needs supplementing and stakeholder consultation may be especially helpful in these cases.

### Casuistry

With deep roots in ancient moral philosophy and modern anti-theoretical bioethics [17], Casuistry uses practical cases with an undisputed solution to solve the moral challenging situation or dilemma in hand. Oriented away from theory or principles and towards the particular, the procedure in Casuistry starts by identifying the structure of the case, i.e., by describing the circumstances (who, what, when, where, how, by what means) and the relevant maxims involved, e.g., “the morals of the story.” Then it compares the case with similar “paradigmatic” cases [10]. Paradigmatic cases are those where a solution is found which is generally accepted. The comparison of cases should reveal the moral maxims at stake and the subsequent practical implications [18]. In HTA, Casuistry can be at play informally, e.g. when referring to solved cases such as coverage decisions, but it can also be more formally applied [19]. As with other approaches in applied ethics, Casuistry has its shortcomings, e.g. potential changes in the value base between past precedents and current cases [18], and it “suffers from the potential limitations of relying on subjective analogic arguments and intuitive judgment about a particular case” [4].

#### Casuistry and the characteristics of complexity

##### Variety of perspectives

Casuistry is well suited to addressing multiple perspectives and goals, as earlier cases may have found (combined or compromised) solutions. The rejection of universal moral norms also fits the need for flexibility in complex interventions. This makes it possible to take into account how socio-cultural, religious etc. circumstances matter in the comparing case, and analyse the similarities with the case under investigation. Moreover, different types of experts may take part in the explication of an ethical problem in Casuistry, which may enable the capturing of possible ethical issues arising from a ‘number of groups or organizational levels targeted by the intervention.’ However, to find cases that join, synthesize, or compromise various perspectives may be challenging. The chosen analogues (paradigmatic cases) instead tend to favour one perspective. E.g., it is difficult to find an analogue to substance abuse that both embraces “victim-of-disease” and “weakness-of-the-will” perspectives. The same challenge occurs when trying to address interconnectedness and interactions between relevant components of health interventions.

##### Indeterminate phenomena

Analogies may be used to conceptualize vague or indefinite phenomena [20, 21]. However, it may be difficult to find analogies that bear sufficient breadth and similarity to the intervention one is assessing. Many and interacting components in complex interventions, may decrease the chances of a “perfect match”, and the choice of an analogue may shortcut or settle important challenges without appropriate consideration. For example, viewing obesity as a metabolic disease may shortcut important psychological and social aspects highly relevant for successful treatment. Hence, there is a chance of “over-simplification” and reducing complex issues to “simple solutions”. On the other hand, it may be possible that two cases are similar regarding moral inferences, even though characteristics of complexity are different. Casuistry does not address potential variation in use or alternative use of interventions.

##### Uncertain causality

Casuistry may be well suited to handle uncertainty in cases where the analogues do so as well. However, Casuistry (in HTA) is not suitable to handle or compensate for emerging uncertainties during the implementation and use of a complex intervention. This is because the complex intervention being assessed may develop differently from the paradigm cases. Although the process in Casuistry can address methodological choices in HTA as well as cherish transparency, this may be masked by the chosen analogue. For example, viewing peptic ulcers as a stress related acid imbalance definitely guided treatment and outcome assessment. However, in the view of present knowledge about helicobacter pylori it is clear that the dominant analogue was limited and limiting. Hence, it can be challenging to fulfil the requested justification of choices in Casuistry due to uncertain causal pathways.

##### Unpredictable outcomes

Some analogues (paradigm cases) may include handling unpredictable outcomes, but whether these are relevant for the given case will inevitably be subject to pure speculations. If one analogy works for handling one outcome that could not be predicted, it is far from clear that it could do the same for another. This means that Casuistry may address unpredictable outcomes, but it may fail as well. Moreover, Casuistry may give the impression that moral problems are always solvable [22]. However, for radically new and emerging technologies with dynamic challenges, such as unpredictable outcomes, this may not be the case.

##### Ethical complexity

Casuistry is excellent for finding solutions to morally challenging problems. However, Casuistry does not provide solutions to genuine paradoxes and aporias. It may find solutions by preferring one perspective or one principle over another, but not by resolving paradoxes. Framing “hearing disability” in the perspective of sign culture does not resolve the tension between the language perspective and the function reduction. Nevertheless, pragmatic (and compromising) solutions in one area may inform potential solutions in other fields.

In summary, Casuistry is well suited for addressing several aspects of complexity as shown in Table 2. However, it may be less suitable for addressing uncertainty, unpredictable outcomes, and ethical complexity. Using it in an interactive way, however, could overcome some of these shortcomings [19].

### Wide Reflective Equilibrium (WRE)

Wide Reflective Equilibrium (WRE) is a coherentist model of moral argumentation [23]. Coherentist, here, is used in contrast to foundational approaches, which assume that there are certain undisputable basic principles from which moral judgments can be derived. Given the—allegedly—undisputable nature of these basic principles, the validity of moral judgments hinges on the validity of the deductive argument. In a coherentist approach, no such assumption is made. Instead, the validity of a moral judgment depends on the coherence (or mutual support) among general moral principle, moral judgment, and background theory. The method has become more widely known since it was used and advocated by John Rawls in his Theory of Justice (1971) [24]. In this work, Rawls tries to argue that his concept of justice as fairness is superior to the utilitarian concept of justice. To do so, he argues that justice as fairness coheres with our considered moral judgments, and is independently supported by background theory concerning human behaviour, such as risk-aversiveness and mutual cooperation. The method has been further elaborated by Norman Daniels [25, 26]. Examples of assessments of healthcare technology where the method of WRE has been used include telemedicine-supported home care [27], genetic engineering [28], and home environments for adults with significant disability [29].

#### Wide Reflective Equilibrium and the characteristics of complexity

##### Variety of perspectives

WRE is a specific type of formal model of moral argumentation. It can be used by an individual person to develop and justify a judgment of the moral acceptability of a specific (healthcare) practice or technology. For instance, to develop an argument to challenge or support prenatal testing for autism. What would be characteristic of the argument is that it claims coherence (and hence mutual support) among the various components (judgment, principle and background theory), each of which may be scrutinized for its relevance and validity separately. However, WRE may also be employed in the context of a moral deliberation with multiple participants. These could be selected to represent a variety of perspectives, e.g. patient, parent, caregiver, healthcare professional, healthcare insurer, etc. In such cases, WRE may be used to structure the development of a collaborative argument, taking into account multiple perspectives on a healthcare technology and any associated differences in judgment of moral propriety [30]. Some stakeholders might, for instance, consider their acceptance of cochlear implants (CI) for deaf children in equilibrium with the open future argument. They might find independent support for their argument from background theory of the association between hearing, cognitive development and socio-emotional development of children [31]. From the perspective of WRE, this might be considered a provisional equilibrium, achieved by a number of stakeholders, which may be challenged by others. The main advantage of WRE would then be, then, to provide structure to the moral argument, allowing participants to explore the coherence among the various elements and the plausibility of the background theory.

##### Indeterminate phenomena

Not infrequently, different interpretations of particular conditions or interventions lie at the basis of ethical debates. In the case of the CI, for instance, a considerable research effort has been directed at assessing the impact of the technology on outcomes such as hearing and the understanding and production of spoken language [3]. However, from the perspective of Deaf Communities, a major concern was the survival of Deaf Culture. From this, it may be inferred that deafness was given a different interpretation. By some, it was considered a medical condition (e.g., due to dysfunctional hair cells in the cochlea). By others, deafness was rather considered a particular way of life (Deafness as culture). WRE can help to identify such indeterminacy, provided that multiple stakeholders have an opportunity to contribute to the deliberation. A critical position toward CI (judgment) could then be related to the idea that it would deprive deaf people from a sense of self-esteem (principle), supported by a background theory on the relation between being part of a culture and well-being. WRE might help participants to use a deliberative process to reach agreement on what interpretations should be taken into account.

##### Uncertain causality

Perceiving ethical issues that are associated with the use of a healthcare technology implies causal attribution: ethical dilemmas are thought to arise from the use of a healthcare technology. However, things will usually be more complex than this. For instance, whether the use of a healthcare technology results in ethical dilemma may, to a large extent, depend on the social context. For instance, in Sweden, the use of CI in deaf children seems to have been far less contentious than in many other countries. This may be related to the fact that in Sweden, an environment of equal opportunities and inclusive disability policies for its citizens was maintained and that in education, a bilingualism method of Swedish Sign Language and spoken Swedish was used consistently in teaching deaf children [32]. WRE may help to identify such instances of more complex, multifactorial causality, especially because it consistently includes background theory in moral arguments. A background theory supporting a critical view of CI would lay out the consequences of CI for Deaf Culture. A more refined background theory would point out that CI is not sufficient cause for a demise of Deaf Culture, and that particular aspects of the social context may be equally important in this respect.

##### Unpredictable outcomes

It is impossible to predict how exactly a particular technology will be used over time. For instance, the history of the CI goes back to the 1950s, when an electrode was inserted in the cochlea of a deaf person for the first time [33]. At the time, it was impossible to tell whether it would evolve into a clinically viable technology. After numerous technical improvements, the technology was initially used in people who had lost hearing later in life. It was only when the technology was used in deaf children, that a fierce debate on its ethical acceptability started. Being a formal method of moral argumentation, of course, WRE does not have anything to offer in terms of a better understanding of technological change. However, this example does show that judgements of the moral acceptability of healthcare technologies should always be considered as provisional and subject to change, notably when the use of these technologies change over time. From the perspective of WRE, this may be interpreted as an instance of a disruption of an existing equilibrium, calling for a renewed debate.

##### Ethical complexity

Probably one of the key causes of ethical complexity is the commitment to a variety of moral values within a community that seem to be in direct opposition and incompatible in concrete cases. Different schools of ethical thought vary in the way they propose to resolve such dilemmas. The case of the CI for deaf children was complex, not least because there seemed to be a conflict between values such as ‘open future’ and cultural diversity. WRE proposes to resolve such value conflicts by trying to discover whether there is differential support for such ‘narrow equilibria’ (between moral judgments and moral principles) from relevant background theories. Clearly, this approach differs substantially from other approaches such as weighing and specification.

In summary, WRE allows an evaluator to take into account the complexity of an intervention and its sequelae, since it explicitly addresses how judgments, principles and background theory cohere with each other. However, to this end, it should be used as a method of moral argumentation in the context of public deliberation, allowing stakeholders to provide arguments from different perspectives.

### Interactive, participatory HTA approaches (iHTA)

Interactive Health Technology Assessment (iHTA) is a specific approach to HTA, involving stakeholders throughout the entire assessment process [34], i.e. people who may experience the consequences of the assessment are involved in defining the research question(s) to be addressed (scoping), in designing the assessment and in the collection and interpretation of the data. The term ‘interactive’ refers to an interaction among the various stakeholders: the explicit objective of the HTA is that stakeholders learn from each other [34]. As such, iHTA is a specific type of frame-reflective analysis [35]: it aims to reconstruct and critically appraise the frames that stakeholders use to interpret the problem and to judge solutions. Typically, such reconstruction of interpretative frames is achieved through semi-structured interviews with stakeholders [36]. Philosophically, iHTA is an approach to HTA which accepts fallibilism without embracing scepticism, and which puts primacy on practice. As such, iHTA can be considered to be firmly rooted in pragmatism [37]. iHTA has been used to evaluate a wide range of technologies, both within [30, 38] and outside the healthcare domain [39].

#### Interactive, participatory HTA approaches (iHTA) and the characteristics of complexity

##### Variety of perspectives

iHTA is pre-eminently suited to take into account a variety of perspectives on a specific problem and their associated (technological) solutions. It acknowledges that ‘Assessment processes are embedded in different sorts of institutional settings, within which scientists, decision-makers, and advocates communicate to define relevant questions for analysis, mobilize certain kinds of experts and expertise, and interpret findings in particular ways’ [40]. One of its key strengths may be that it helps to reveal that problems may be defined quite differently by various stakeholders; that stakeholders consider a different approach to resolve the problem, and that they employ different standards for evaluation. To increase a sense of ownership and to increase the prospects for implementing strategies for resolution, it is imperative that such differences are identified early on in the assessment process. Collaboratively, participants should decide on relevant issues to address and feasible approaches of inquiry. In the case of the cochlear implant (CI) for deaf children, stakeholders radically differed in terms of the issues of concern. Whereas healthcare providers tended to emphasize research on the production and understanding of speech, parents of deaf children also desired to know whether CI might improve the reading skills of their child. Representatives of Deaf Organizations wanted to know how a CI might affect the self-esteem of the recipient at the time they would become adolescent. One of the key challenges in iHTA may be to organize the assessment process in such a way, that the concerns of the various perspectives are taken seriously and expressed in the actual inquiry. iHTA may be an effective means to achieve this goal.

##### Indeterminate phenomena

One of the key pitfalls in HTA may be to take a (health) problem, its associated solution and the evaluative framework as given. However, problems do not exist independently of the interpretive frames that are being employed in particular social contexts, nor do their solutions. The nature of the problems that are posed by deafness may differ, depending on whose perspective is taken, and so may their solutions. One of the key threats to the integrity of HTA is to uncritically adopt a dominant way of framing an issue. iHTA can help to prevent this from happening. In iHTA, a technology and its use are not considered as fixed, or given. Indeed, one of the key objectives of the assessment is to discover, collaboratively, what the optimal use of a technology in a particular context might be. In this sense, iHTA is formative, rather than summative [41]. However, one challenge is to have all stakeholders equally involved in the continuous re-definition of core concepts. For instance, in the case of the CI, this would mean that the objective of the HTA is not to establish the value of the technology. Rather, the objective is to define under what conditions the CI might be a valuable option for all stakeholders involved. This gives rise to an entirely different line of inquiry.

##### Uncertain causality

Stakeholders can, and often do, disagree on the likelihood that particular consequences will emerge, following the use of a technology. iHTA can serve as a useful approach to uncover such divergent views, and to critically appraise their empirical and theoretical foundations. The reason for this is that an important element in iHTA is the reconstruction of interpretative frames that stakeholders bring to the inquiry. These include judgments of solutions, problem definition, background theory, and normative preferences. Differences in judgment of solutions (their effectiveness, appropriateness, efficiency, etc.) can often be traced back to differences in background theory [42]. Once these have been made explicit, their validity can be more readily assessed. In the case of the CI, for instance, some parents of deaf children preferred to postpone a decision on CI until their child was old enough to be involved in the decision. Although understandable, it should be pointed out to such parents that the outcome is likely to subside with increasing age at implantation. The outcome of CI critically depends on a host of factors, including the age at implantation and the communication of the parents with the child. Addressing the underlying mechanisms of child development may help the various stakeholders in to understand these issues better.

##### Unpredictable outcomes

Adopting an Interactive approach to HTA (iHTA) will not allow for a more accurate prediction of future outcomes. However, it will usually lead to a wider range of relevant potential outcomes. As indicated above, in the case of the CI for deaf children, there was a strong focus on the impact of the technology on hearing and the understanding and production of spoken language. However, the (hearing) parents of deaf children pointed out that for them, the impact of the technology on the reading proficiency of their child was hugely important [30]. Thus, an interactive approach to HTA is likely to increase the range of outcomes that are taken into account.

##### Ethical complexity

One of the key advantages of an interactive approach to HTA may be that it allows for a better appreciation of the complexity of the ethical issues associated with the (use of) a technology. The reason for this is that the technology is viewed from a range of perspectives, by stakeholders who bring different values to the inquiry. For instance, in the case of the CI, some stakeholders seemed to reason from the open-future argument, while others put more emphasis on the value of cultural diversity. Both represent defensible moral positions, generally. The challenge is to acknowledge such complexity and to devise acceptable solutions in concrete cases. iHTA, of itself, does not provide the tools to normatively assess the validity of ethical arguments. For this, methods of moral argumentation such as Wide Reflective Equilibrium or Casuistry may be used in the context of an iHTA [30].

In summary, iHTA offers a way of systematically involving multiple stakeholders, creating opportunities for mutual learning and improved understanding. iHTA’s emphasis is on procedural justice. Arguably, its main added value will be in cases where quite divergent views exist among stakeholders on the nature of the problem to be solved and the most promising routes for its resolution. This, in itself, may be considered to be an indication of complexity. In principle, the method is never ending.

### The HTA Core Model 3.0®

The HTA Core Model 3.0® has been developed in the course of the European network for Health Technology Assessment Joint Action 2 (EUnetHTA JA2) [43]. It consists of nine domains, among which is the domain “ethical analysis”. The “ethical analysis” domain is divided into six topics; three of them (Beneficence/Non-maleficence; Autonomy; Justice and Equity) are directly related to the Principlist approach to bioethics (see paragraph on Principlism). The other three topics are respect for persons, legislation, and ethical consequences of the HTA. Each topic consists of two to four questions, adding up to nineteen assessment “issues”. Authors of HTAs are encouraged to start by gathering information on ethical issues using systematic literature searches, professional guidelines, and the stakeholder views. In a second phase, it is suggested that users choose from different methods that have been assembled by a working group of the International Network of Agencies for Health Technology Assessment (INAHTA) [44]. The choice of the method should depend on factors such as the type of technology, the role and authority of the HTA organisation, the time and resources available and the expertise with ethical analysis available within the organization. For a comprehensive analysis, it is recommended that more than a single method is applied, and experts in ethical analyses are involved, in addition to other scientists and clinicians [43]. “The need, weight and complexity placed on the ethical analysis can differ between technologies, and for the same technology for a rapid and full assessment depending on the purpose and context of its use” [43].

Beyond the ethical domain, the HTA Core Model® introduces ethics as a wider principle and provides a list of ethical issues that should characterize each HTA. These comprise e.g., stakeholder involvement, “morally relevant reasons for performing/not performing a HTA” on specific topics, or the description of the interests of the manufacturer. It acknowledges that the HTA-process itself is characterized by value judgments and that these value judgments should be made explicit. Examples for the ethical analysis guided by the HTA Core Model® are an HTA on Multi-Slice Computed Tomography (MSCT) [45] an HTA on Drug Eluting Stents (DES) [46] and an HTA on Abdominal Aorta Aneurism Screening (AAA-screening) [47].

#### The ethical domain in the HTA Core Model 3.0® and the characteristics of complexity

##### Variety of perspectives

The HTA Core Model® assesses the implementation of a health technology from the perspective of the prevailing societal values and it looks at the norms and values that the technology itself constructs. It also assesses the ethical issues related to performing the HTA. Assessment involves explicitly looking at the interests of different stakeholders at various levels; among them patients, relatives, and society. Assessment involves developing a table to synthesize the evidence consequences of implementing or withdrawing the intervention for the patient, family/important others, health care providers, society, and others. The variety of perspectives is also reflected in the iterative process of the analysis, including the involvement of different experts. However, the active involvement of stakeholders, patients and the public seems to be difficult because of time and resource constrains [45, 46] as well as the level of the analysis [47]: “The general approach on the EU level has to be put into operation at the local level. This could be done using an Interactive participatory approach to HTA”. The AAA-screening example identified the potential for different perspectives at the local level with regard to issues of professional autonomy. However, differences between professionals and other participants are neither part of the analysis, nor related to one another.

##### Indeterminate phenomena

Defining the technology as well as the target group is not directly addressed in the ethical domain of the HTA Core Model®. Both are part of the “Description and technical characteristics of technology” domain. Moral challenges linked with the definition of the intervention would be part of the iterative ethical reflection during the assessment of this domain. The domain asks for a detailed description of the intervention’s components including a glossary and information about the “scientifically proven versus suspected mechanisms of action”. This is used to ensure transparency and clarity about the technology.

##### Uncertain causality

The HTA Core Model® encourages the user to undertake the ethical analysis throughout the HTA-process rather than seeing it as a “one session” task. Multiple components and difficulties in determining the intervention and target group lead to uncertain causal tracks. The ethics domain contains this up to a certain point, e.g. asking for intended and unintended consequences of the implementation or use of the technology and suggesting the involvement of different stakeholders and the public from the very beginning of the HTA. Furthermore the HTA Core Model® addresses the context and non-technology-specific values such as autonomy, dignity or integrity as well as professional values and traditional roles.

##### Unpredictable outcomes

Through a set of suggested issues, the approach encourages the user to think about a wide spectrum of possible outcomes and to link these (iteratively) with outcome measures identified in the other domains of the HTA Core Model®. The downside of such an approach is that users might apply it as a finite list of issues ignoring other issues that are not explicitly mentioned. In the HTA Core Model® the assessment questions are chosen at the beginning of the ethical analysis. In the example of the AAA-screening six assessment questions were marked as not relevant. Among them were questions related to challenges of patient autonomy, potential benefits and harms for other stakeholders.

One of the issues under the topic “beneficence/non-maleficence” addresses “hidden or unintended consequences of the technology and its applications for different stakeholders”, and the ethical dimension of identifying outcome measures is stressed explicitly through the topic “Ethical consequences of the HTA”. Examples are incidental diagnoses related with the MSCT [45] or “off-label”-use in the example of DES [46]. In the ethical reflection of the AAA-Screening [47] this question was classified as “not relevant” explaining that “hidden or unintended consequences can only be considered on the local level”.

##### Ethical complexity

Contradictions between basic ethical principles are not explicitly mentioned in the HTA Core Model®. The approach acknowledges that ethical analysis, although being a separate domain, is closely interrelated with aspects such as safety, clinical effectiveness or cost-effectiveness. Collaboration between ethical experts and stakeholders (e.g. clinicians) is suggested, which could contribute to identify ethical complexity as well as transparency. Stakeholder involvement may reveal fundamental values at stake and possible contradicting principles and values.

In summary, the HTA Core Model® offers a structured and transparent method for the ethical assessment of technologies. The HTA Core Model® essentially follows a Principlist approach and the use of tables as a basic structure for analysis might impede the application of approaches reflecting the consistency of ethical arguments and background theories “without prescribing which facts, arguments or principles are prima facie relevant” (e.g., Coherent Analysis). A strong focus on the assessment tables could mean that important aspects of a specific technology are overlooked. The HTA Core Model® addresses many of the complexity aspects, although not always in a systematic way. The way methodological approaches are linked with the assessment tables remains open. Depending on the methodological approach chosen the complexity of a health technology will be more or less taken into account.

### The Socratic approach

The Socratic approach is axiological as it studies values. The aim is to uncover and highlight the values, norms and ethical challenges that are relevant for the health intervention, the HTA process, as well as for the decision making process. The Socratic approach consists of six steps and seven basic morally relevant questions, which are further specified in thirty-three explanatory and guiding questions [7]. For clarity, we have provided the number of steps and questions in parentheses when comparing the Socratic approach with the complexity characteristics below. The exact phrasing of steps and questions can be found in the revised version of the approach by Hofmann et al [7].

The Socratic approach has been applied in a range of assessments, e.g. of Bariatric surgery [48], Autologous stem cell transplantation [49] and Welfare technology [50]. These interventions carry some of the complexity characteristics and indicate that the approach is feasible for the assessment of complex health interventions.

#### The Socratic approach and the characteristics of complexity

##### Variety of perspectives

Identifying persons, groups, and stakeholders is a separate preparatory step (2) in this approach. In addition, the stakeholders respective perspectives, interest, values and intention is the focus of many of the questions from the list, e.g. questions about the *interests* of users (Q19), producers (Q21), persons participating in the HTA (Q28), and autonomy of the professionals (Q20). Revealing the purpose and background for the HTA (step 1) may also provide information on stakeholder interests. Additionally, a reflexive dialogue with stakeholders is recommended.

The question of normative implications of interactions/relationship between agents is indirectly and partly covered by the question of relationship between patients and health care professionals, and within the latter group (Q12). Similarly, the general issue of interactions between numerous (human and non-human) components are briefly addressed (in Q16), but this may not be ethically relevant unless they influence other characteristics of complexity (e.g. outcome uncertainty). Many characteristics of interactions between the intervention as a whole and people/society are covered, e.g. the questions about whether use of the technology will change the patient role (Q3).

Issues of control and decision-making and related responsibilities (as part of the technology itself) are not directly addressed in this approach, though it is touched upon in the question about impact of the technology on professional’s autonomy (Q20). The obligations of people doing the HTA are more detailed as indicated in Q23 – 31.

##### Indeterminate phenomena

Various understandings and interpretations of the interventions can be explored as part of the initial identification of the purpose of the health intervention and background for the HTA (step 1). Additionally, the undefined nature of the complex intervention(s) may be further illuminated by the recommended stakeholder involvement. It seems paradoxical that the scoping process and selection of experts/stakeholders to include (Q26) is ever more important when the concept and understanding of the intervention is wide and unclear. Indeed, at the same time, this vagueness may confine the fraction of experts that it is possible to include. For example, in the case of Welfare technology, which covers a heterogeneous group of technologies [50], the number of possible different experts/stakeholders may be too large to include all/a reasonable fraction.

The approach contains questions about moral issues related to the characteristics of the technology (Q14-17), and about the health condition and the target population(s) (Q1 – 4). These may reveal indeterminacy and contradictions, e.g. regarding purpose of the intervention or the status of the disease. Consequently questions about justification of flexibility, tailoring, alternative and unexpected use of the intervention can be addressed.

##### Uncertain causality

The Socratic approach includes several questions about morally relevant methodological choices in the HTA. For example, it includes, questions about choice of end points, cut-off values and outcome measures (Q23), selection (criteria) of studies to include in the HTA (Q24), the choices in the HTA process from planning (scoping, expert group selection) to presenting results (Q26), specific presumptions and methodological choices in the economical part of the assessment (Q27), and timing of the assessment (Q29). Such questions can illuminate how appropriate the HTA is for taking into account the uncertainties in causal pathway between intervention and outcome, and pave the way for improvements. Using the approach in an integrative manner will increase transparency of methodological choices and their justification.

The symbolic value of the intervention is addressed (Q15) and the impact of context on outcome of analyses is recognized. However, the relevance of these factors may not be directly linked to their possible impact on causal pathway between intervention and outcome. Symbolic value and context may be ethically relevant for other reasons, e.g. related to prestige and stigmatisation. A systematic review of autologous stem cell transplantation (ASCT) in advanced breast cancer, using the Socratic approach, illustrates how this intervention became loaded with the symbolic value of “dissemination of phase II trial results, coverage without evidence, falsified data, high costs to the public and considerable, unnecessary harm to patients” [49]. In this case, the negative impact on recruiting patients and the doctor-patient relationship was reported.

##### Unpredictable outcomes

Even if the variety of outcomes (and for whom) is not addressed specifically, many moral question about (potential) outcomes or consequences are addressed: e.g. change of patient role (Q3), effects on the distribution of health care (Q7), consequences (benefits and harms) (Q8), moral obligations (Q10), change of relationships (Q12), alteration of professional autonomy (Q20).

The uncertainties (be it risk, uncertainty, ignorance, or indeterminacy/ambiguity) in outcomes (type and size) are not addressed as such, e.g. if some kind of uncertainty itself may infer harm, challenge autonomy etc.

##### Ethical complexity

The aim of the approach is to highlight (underlying) values, viewpoints and arguments regarding an intervention that are important for decision-making. This is achieved by comprehensiveness in the normative issues addressed [49] (also including socio-cultural and legal issues) and moral pluralism (incorporation of Deontology, Utilitarianism, Principlism, Casuistry, Virtue ethics) [7]. Altogether this should make a solid basis for the additional step that may be required in cases of complex interventions: to elucidate possible contradictions and clarify their nature and solvability.

Whether the Socratic approach is developed for people with or without philosophical qualification is not clear. The method may be applied by HTA experts without such qualifications, but not all questions are easy to address [7]. For application on complex interventions, the approach may either be applied by trained philosophers/ethicists, or be developed to include additional explanations and guidance.

The thoroughness described above also makes the approach appropriate for revealing potential fundamental values at stake, particularly if is applied in a reflexive dialogue with stakeholders as recommended. However, the approach’s contribution to handling conflicting concerns may be limited, or in fact be beyond its scope, as it is modelled for assessments and not for appraisal. It is clearly stated that the aim and scope of the approach is to provide a descriptive assessment and to leave the appraisal to those responsible for decision-making, but it is recognized that more guidance on how to balance benefits and harms in a systematic way would be valuable [7].

The Socratic approach aims at comprehensiveness in documentation and reporting of the assessment process (the six steps) [7], but this isn’t always apparent in publications applying the method. The most recent version also includes additional recommendation e.g. regarding information retrieval and selection, and quality checks of extracted data. Transparency in all steps is highlighted, not least about important stakeholders’ perspectives.

In summary, the Socratic approach meets the complexity criteria fairly well. The most important points for improving its applicability are: to address questions of decision making and responsibility; to address the moral impact of indeterminacy and uncertain of outcome and finally, to offer some guidance in how to balance conflicting concerns.

## Conclusion

This study shows how the applicability for addressing ethical issues in HTA of complex health interventions differs between ethical approaches. The approaches are assessed according to a set of five characteristics of complex interventions, which in sum reflect their overall ability to address ethical analysis for these kinds of technologies. In general, processual approaches, such as Interactive, participatory HTA (iHTA), seem to be able to handle most aspects of complexity. The interactive and flexible nature of this approach makes it suitable for handling the unpredictability embedded in complex interventions. The flexibility and openness for different perspectives may also explain why Wide Reflective Equilibrium seems better suited for complex health interventions than more rigid general approaches, such as Principlism and Casuistry. The two latter approaches should be supplemented with additional perspectives and/or applied in a more interactive way, when assessing complex health interventions. The two other HTA specific approaches, The HTA Core Model® and the Socratic approach, also seem fairly applicable for complex health interventions, which should not be surprising as they include various ethical approaches and perspectives.

We believe that our work will be helpful when choosing and applying appropriate approaches for ethical analyses in HTA. However, one should be aware of other important factors that may influence the choice and application of an ethical approach, such as local HTA agencies resources, possible locally developed guidelines and whether the aim of the HTA is assessment or appraisal. We also want to emphasise that all ethical approaches have limitations, and challenges associated with conflicting values, interests and consideration should always be expected in ethical analyses. Our study can be helpful in preparing for some ethical assessment challenges embedded in complex interventions, and increases the awareness of ethical aspects of complex health interventions in general.

## Footnotes

1 *Health interventions* seem to be the most commonly used term in the complexity literature, and *intervention* is a catch-all term, conflating quite separate initiatives like a clinical treatment, a health care program, health services delivery etc. [53]. Hence we understand and use this term broadly as equivalent to the term *health care technology*, which is also in accordance with the INAHTAs definition of *health technology*: “Any *intervention* that may be used to promote health, to prevent, diagnose or treat disease or for rehabilitation or long-term care. This includes the pharmaceuticals, devices, procedures and organizational systems used in health care” (our italics) [54].

2 The term ‘complex’ refers to integrations between connected things [55], which are causing unpredictability.

3 The range of disciplines in (bio)medical ethics includes for example (moral) philosophy, anthropology, economics, epidemiology, health services research, history, law, literature, medicine, nursing, moral and social psychology, social sciences, and theology.

4 EUnetHTA was established first as a project, and later as a network, with the aim of to support “collaboration between European HTA organisations that brings added value at the European, national and regional level through: facilitating efficient use of resources available for HTA, creating a sustainable system of HTA knowledge sharing, and promoting good practice in HTA methods and processes” (http://www.eunethta.eu/)

5 One reason for excluding ethical approaches rarely or never used in HTA was the limited information available for the analysis. Furthermore, the focus is on ethical approaches, which means that approaches for obtaining and synthesising ethical data are not included.

6 The complexity characteristics described in Table 1 are not mutually exclusive. Overlapping between the characteristics could be expected due to the nature of complexity.

7 Lysdahl KB, Hofmann B. Complex interventions: Characteristics relevant for ethical analysis in health technology assessment. Submitted manuscript.

8 It would be valuable to apply a wide range of ethical approaches for one and the same complex intervention and compare the outcomes, but this would be highly context dependent. To apply all ethical approaches for the same complex interventions, in the same context, would amend this, but it would be highly resource demanding.
